# Legalization of Anatomy

**Published:** 1847-09

**Authors:** 


*Legalization of Anatomy.*—The National Convention, at their late session, passed a resolution recommending to the profession in states wherein laws legalizing the practical study of anatomy do not exist, to unite in endeavoring to procure the passage of such laws. In accordance with this resolution, the Erie County Medical Society, at the semi-annual meeting in June last, appointed a committee, of which; Dr. G. N. Burwell of this city is chairman, to correspond with other societies, and with members of the profession in this state on the subject. We know not whether any steps have, as yet, been taken toward carrying out the purpose of the resolution. We hope, however, that the approaching legislative session will not be allowed to pass by, without some means being taken to bring the matter before it. It is, of course, quite unnecessary to say a word to Medical men of the propriety, and great importance of such laws. The only questions are, can they be procured: is the state of public sentiment prepared for them? From what reflection and observation we have bestowed on the subject, we are convinced that these questions may be answered affirmatively. We believe that if the subject be fairly submitted to the Legislature, and the public, the policy in various points of view of providing legally for dissections will be appreciated, and acted upon. We have not time nor space to extend our remarks in this No., and we will, for the present, content ourselves with earnestly inviting our brethren throughout this state to give the matter thought, and, if they agree with us that the present is a proper period for effort, their co-operation in such measures as shall be neces sary to effect the object. We would suggest that they who are disposed to take an interest in the matter, draw up petitions to the Legislature, to be signed, not by Physicians only, but by intelligent persons of all classes. We feel assured that the larger portion of all communities will cheerfully unite in an application for the legalization of Anatomy, especially if it be explained to them that it will not only promote their interests by its influence on medical and surgical knowledge, but will afford the best security against outrages, which, until dissections are provided for by law, will occasionally occur.

